# Evaluating malaria elimination strategies among military forces in Cambodia: a multi-arm clinical trial comparing monthly prophylaxis and focused screening and treatment

**DOI:** 10.1186/s12879-025-12207-4

**Published:** 2025-12-12

**Authors:** Mariusz Wojnarski, Sidhartha Chaudhury, Threechada Boonchan, Rathvicheth Bun, Soklyda Chann, Panita Gosi, Kin Soveasna, Sokhun Song, Nillawan Buathong, Mali Ittiverakul, Sabaithip Sriwichai, Montri Arsanok, Worachet Kuntawunginn, Piyaporn Saingam, Chaiyaporn Chaisatit, Alongkot Ponlawat, Thanyalak Fansiri, Pattaraporn Vanachayangkul, Boonsong Jaichapor, Muth Sinoun, Char Meng Chuor, Thay Kheangheng, Mary So, Elizabeth Wanja, Silas Davidson, Michele Spring, Huy Rekol, Lek Dysoley, Kong Saly, Jeffrey R. Livezey, Jessica T. Lin, Philip L. Smith, Prom Satharath, Jessica E. Manning, Somethy Sok, David L. Saunders

**Affiliations:** 1 Walter Reed Army Institute of Research - Armed Forces Research Institute of Medical Sciences, Bangkok, Thailand; 2 Department of Health, Ministry of National Defense, Phnom Penh, Cambodia; 3 National Center for Parasitology, Entomology and Malaria Control, Phnom Penh, Cambodia; 4 University of North Carolina School of Medicine, Chapel Hill, NC USA; 5 Uniformed Services University School of Medicine, Bethesda, MD USA; 6 International Center of Excellence in Research, National Institute of Allergy and Infectious Disease, National Institutes of Health, Phnom Penh, Cambodia; 7 Laboratory of Malaria and Vector Research, NIH, Bethesda, MD USA; 8 School of Public Health, National Institute of Public Health, Phnom Penh, Cambodia; 9 Defense Health Agency, Falls Church, VA USA; 10 Sanofi-Pasteur, Bethesda, MD USA

**Keywords:** Malaria, Elimination, Mass drug administration, Prophylaxis, G6PD

## Abstract

**Background:**

Identifying effective malaria elimination strategies for remote regions and highly mobile populations in Southeast Asia is challenging given limited resources. In this clinical trial, two malaria elimination strategies were evaluated in partnership with the Royal Cambodian Armed Forces - monthly malaria prophylaxis (MMP) and focused screening and treatment (FSAT).

**Methods:**

Eight military cohorts (1,050 volunteers total) along the Cambodian-Thai border were randomly assigned to three months of either MMP or FSAT (four cohorts in each treatment arm) with monthly malaria testing for six months. Cohorts were further randomly assigned to receive either permethrin treated (ITU) or sham-treated clothing (sITU). Volunteers in MMP cohort were given three monthly three-day doses dihydroartemisinin-piperaquine (DP) along with a ‘universal’ low-dose weekly regimen of 22.5 mg primaquine for 12 weeks, intended for use regardless of body weight or G6PD status. Volunteers in FSAT cohort were treated with appropriate first-line antimalarials if they tested positive for malaria.

**Results:**

*Plasmodium falciparum* (*Pf*) positivity in MMP cohorts was reduced by 90% (10% at enrollment to 1% at six months; absolute risk reduction (ARR) 9%) at 6 months. However, 32% of *Pf* cases treated with DP as MMP at baseline recrudesced, requiring rescue treatment at 1 month with artesunate-mefloquine. *Pf* positivity in FSAT cohorts declined 66% over six months (7.6% to 2.7%; ARR 4.9%). MMP reduced *P. vivax* (*Pv*) positivity from 9% to 0% at three months, but *Pv* rebounded to 6.7% at six months. FSAT failed to significantly reduce *Pv* positivity during the study. The 22.5 mg weekly primaquine MMP regimen was safe, even for the 15% of volunteers with G6PD-deficiency. Those wearing ITU had additional *Pv* parasitemia reductions compared to sITU in the FSAT but not MMP cohorts.

**Conclusions:**

MMP was safe, and superior to FSAT, suggesting greater utility to achieve malaria elimination in Cambodia. Low dose (22.5 mg) weekly primaquine was a safe adjunct in this setting, even for those with G6PD-deficiency. Permethrin-treated clothing further reduced *Pv* parasitemia for FSAT but not MMP. We observed that MMP may be more easily scaled to eliminate malaria and that the military may provide substantial support for regional elimination efforts.

**Trial registration:**

The study was registered on https://clinicaltrials.gov/ on 13-01-2016 prior to enrollment (NCT02653898).

**Supplementary Information:**

The online version contains supplementary material available at 10.1186/s12879-025-12207-4.

## Introduction

Malaria caused by *Plasmodium falciparum* (*Pf*) and *Plasmodium vivax* (*Pv*) has historically been a major public health threat in Southeast Asia with millions of cases annually. Cambodia, in particular, was a hotspot for the emergence of multi-drug resistant (MDR) *Pf*, where declining efficacy of artemisinin combination therapies was observed within only a few years of their introduction as first-line therapies [1–4]. In 2018, ASEAN leaders committed to a goal of regional malaria elimination of *Pf* by 2025 and *Pv* by 2030 [5–7]. Since then, intensive containment and control efforts have substantially reduced malaria prevalence in Southeast Asia [8], with over 90% reduction in Cambodia from 2010 to 2020 [7], with similar reductions in neighboring Laos and Vietnam. Likewise, peninsular Malaysia and most of the Philippines have recently achieved complete or near-complete malaria elimination [9]. However, *Pv* and cases of MDR *Pf* continue to persist in border areas of Cambodia and Thailand [10], and endemic malaria prevalence is still high in Myanmar, areas of Thailand along the Myanmar border, Palawan in the Philippines, and East Papua and Papua in Indonesia [8]. Malaria elimination strategies are now focused on aggressive efforts to reduce incidence rates in remote areas and high-risk populations [7]. These populations include mobile populations with occupational exposures occurring in forested areas such as the military [11, 12] and populations residing or working in forested border areas [13, 14]. Currently multiple approaches for targeting high-risk populations are being explored, many requiring further evaluation [15].

Two approaches are typically proposed for malaria elimination in this region. The first is ‘mass drug administration’ (MDA) and the second is ‘focused screening and treatment’ (FSAT) where entire communities are evaluated and positive members receive treatment. MDA involves mandatory treatment ranging from focal areas of outbreak to entire endemic communities. While a large number of MDA studies have been conducted over the past 70 years, assessment of their efficacy have often been complicated by the lack of well-defined objectives and varying study designs [16]. Prior studies identified factors associated with MDA success, including achieving at least 80% population coverage, directly observed treatment (DOT), strong community engagement, and the use of 8-aminoquinolines (in *Pv* transmission settings) [17, 18], and the use of repeated administrations in high transmission areas [19]. However, there are ethical concerns with the use of compulsory anti-malarial treatment courses with associated side-effects in healthy individuals, particularly in low-transmission settings. The FSAT approach, although it has significant logistical challenges to implementation [20], is thought to be a more ethical alternative [21]. The FSAT and MDA approaches have not been evaluated head-to-head in Cambodia, and there have been few studies conducted in hard-to-reach mobile populations, particularly the military [22]. Furthermore, despite widespread use of malaria chemoprophylaxis by Western militaries deploying to malaria endemic areas, this approach has not been systematically evaluated nor widely adopted by militaries in Southeast Asia.

Diagnostic challenges also influence potential approaches. Rapid diagnostic tests (RDTs) are a practical option to screen large populations but their low sensitivity limits their ability to identify individuals with subclinical infections and low parasitemia in areas approaching malaria elimination [19, 23, 24]. While PCR offers high sensitivity and specificity, it is expensive, difficult to implement in austere locations, and does not detect the latent stages of *Pv* [25]. Even without PCR, the amount of laboratory support and follow-up required to perform adequate systematic FSAT on even a monthly basis is significant [20].

The optimal approach for malaria elimination is likely to include vector control measures in addition to drug therapy. Vector control measures such as indoor residual insecticide spraying and the use of insecticide treated nets have been widely employed in Cambodia. However, the efficacy of vector interventions suitable for militaries, including use of insecticide-impregnated uniforms and various forms of personal and spatial repellants, remain relatively untested in field settings, particularly against disease endpoints in clinical trials. Permethrin is the most common insecticide used for treating clothing. Few prior studies evaluating permethrin-treated uniforms in preventing malaria did find statistically significant reductions in malaria in both civilian [26] and military populations [27] with a duration of efficacy estimated to be at least 2–3 months. These studies were conducted in the absence of chemoprophylaxis and suggest that insecticide-treated uniforms can play a significant role in malaria prevention. Further studies on the efficacy of insecticide-treated uniforms and topical repellents on malaria infection rates in high-transmission areas are on-going [28]. Use of insecticide-treated uniforms is widespread in many Western militaries when deploying to regions with high prevalence for arthropod-borne diseases yet there is scarcity of recent field data on its efficacy. 

The present study aimed to answer some of the key questions outlined above to define optimal approaches to malaria elimination. The Walter Reed Army Institute of Research – Armed Forces Research Institute of Medical Sciences (WRAIR-AFRIMS) conducted a multi-arm interventional malaria elimination study in partnership with the Royal Cambodian Armed Forces (RCAF) and National Center for Parasitology Entomology and Malaria Control (CNM) in Cambodia. The study aimed to quantify the relative safety and effectiveness of two major interventional approaches to malaria elimination in this region: (1) monthly malaria prophylaxis (MMP) using three monthly three-day fixed-dose treatment courses of dihydroartemisinin-piperaquine (DP) and a weekly fixed low dose of 22.5 mg of primaquine, and (2) focused screening and treatment (FSAT) of only those found to be either microscopy or PCR malaria positive using the current first-line antimalarials recommended for *Pf* and *Pv* respectively. The fixed-dose DP regimen and fixed low-dose PQ regimen for MMP is intended to be used as a ‘universal’ G6PD-safe regimen in a mass drug administration settings with remote or highly mobile populations where individualized dosing, G6PD testing, and routine medical monitoring for risks such as hemolysis may not be feasible. The additive effects of insecticide-treated uniforms on malaria prevalence were also assessed by comparing to sham-treated uniforms or clothing to prevent infectious mosquito bites.

## Materials and methods

A multi-arm, open label interventional study was conducted in eight military residential cohorts on the Cambodia-Thai border to compare the effectiveness of the MMP and FSAT elimination approaches, as described previously [29]. Each cohort included approximately 120 individuals and the eight cohorts were split into four MMP cohorts and four FSAT cohorts. In FSAT cohorts, malaria was diagnosed using either microscopy or PCR, with those found positive were treated with currently recommended medications according to national treatment guidelines. The MMP cohorts received monthly prophylaxis using DP along with 12 weekly doses of low-dose PQ. Cohorts were stratified a priori based on low or high malaria transmission and sub-randomized to wear of permethrin (ITU) or sham-treated (sITU) uniforms or clothing while in forested areas. To execute the study, the WRAIR-AFRIMS research team worked closely with RCAF and CNM to create a scalable military malaria elimination unit at the provincial level that relied on medical personnel stationed with cohorts to monitor disease and provide DOT. This unit included 187 RCAF personnel trained and supplied by the research team, including 170 medics assigned to individual units responsible for day-to-day treatment and follow-up during the study.

### Study population

The study was carried out with eight military cohorts stationed in Cambodia in border region with Thailand. The study sites were largely, but not entirely isolated from the local civilian population and no coordinated population-level malaria control measures were implemented in those regions at the time of the study. Routine malaria control measures in this population include the use of topical repellents and insecticide-treated bed nets. Inclusion criteria included: (1) Volunteers 18–65 years of age and their dependents *≥* 2 years of age, to include military and eligible Cambodian civilians eligible for care at an RCAF facility; (2) Able to give informed consent/assent; (3) Residing in the selected study areas, (4) Available for monthly follow-up for the 6-month study duration; (5) Agreed not to seek initial outside medical care for febrile illness unless referred by the study team; and (6) Authorized by the local Commander to participate in the study if on active military duty.

Exclusion criteria included: (1) Known allergic reaction or contraindication to interventions to be used to include DP, artesunate-mefloquine (ASMQ), or PQ; (2) Pregnant or lactating females, and females of childbearing age unwilling to use acceptable forms of contraception during the study. Study assessments took place in RCAF facilities near the Cambodia-Thai border.

### Cohort randomization, allocation, and blinding

**Fig. 1:**
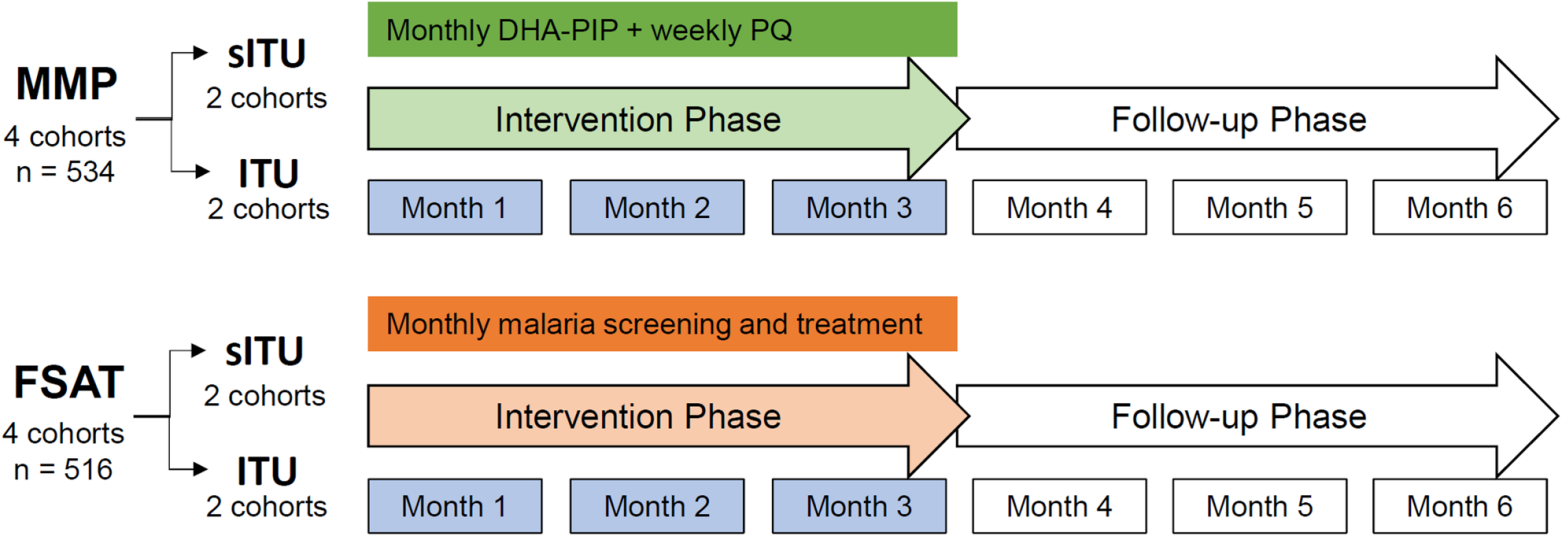
Study design and timeline. All volunteers were screened for malaria with monthly malaria RDT, microscopy and PCR testing. During the three-month intervention phase, volunteers in the MMP treatment arm received regularly scheduled treatment with a full monthly DP treatment course and a weekly dose of 22.5 mg primaquine regardless of malaria positivity for a total dose of 270 mg. Volunteers in the FSAT arm only received treatment if found to be malaria-positive by microscopy or PCR. The treatment arms were further subdivided into those that received insecticide-treated uniforms (ITUs) and sham-treated uniforms (sITUs). All volunteers in the study were screened monthly for three additional months or when symptomatic with RDT, PCR and microscopy and treated for malaria if positive. Treatment with standard-of-care medications was given if positive by microscopy or PCR within 24–48 h. Standard doses of PQ were used per national treatment guidelines in the FSAT arm

In this parallel assignment clinical trial, eight community cohorts were randomized 1:1 allocation to either MMP or FSAT cohorts, using time and region-blocked randomization carried out by the WRAIR-AFRIMS team. The eight community cohorts were selected such that four were from regions with relatively high malaria incidence rate (high endemicity) and four were from regions with relatively low malaria incidence rate (low endemicity), based on internal RCAF medical data on malaria incidence. This ensured that randomized cohorts were geographically distinct, and that treatment (MMP vs. FSAT) and uniform conditions (ITU vs. sITU) were evenly distributed among high and low transmission areas. Volunteers meeting enrollment criteria received either ITUs or sITUs as appropriate based on their cohort. Clothing was treated according to cohort assignment in single-blind fashion with the volunteer, but not the investigators, blinded to assignment. Diagnostic microscopists were blinded to each other’s readings and to study arm assignments. The study design in shown in Fig. 1.

### Recruitment

Sites selected were based at military units willing to participate in the study and authorized to do so by their respective Command authorities. Authorization was granted on a collective basis to military personnel at each participating cohort prior to selection. Individuals at each site were enrolled based on willingness to participate, and authorization for military personnel by their supervisor. However, the Chain of Command was not involved in the enrollment process nor permitted to encourage or order soldiers to participate in the study. Independent civilian ombudsman were present during consent and enrollment to minimize undue influence among participants.

### Study procedures

Following informed consent or parental consent with assent by minors, all subjects underwent baseline assessments including a history and physical exam, complete blood count, and malaria diagnostic testing. Glucose 6-phosphate dehydrogenase (G6PD)-deficiency screening was assessed using FDA-approved quantitative testing methods (Trinity Biotech, Ireland). Malaria screening included a rapid test (SD BIOLINE Malaria Ag *Pf/Pv* malaria antigen or MALACHECK Ag pLDH/HRP2), microscopy and molecular testing with species-specific PCR using previously described methods [29]. A threshold of < 35 PCR replication cycles was used to define positive results for malaria. The study consisted of a three-month intervention period with MMP or FSAT, followed by a three-month follow-up period, during which all volunteers continued monthly screening and treatment. PCR was the definitive diagnostic for defining malaria cases for the purpose of data analysis due to its high sensitivity and specificity for both *Pf* and *Pv*. However, treatment decisions were made based on a positive result by either microscopy or PCR to ensure that treatment was not delayed due to any delays in obtaining PCR results. Allowance of treatment for all patients who were malaria positive on microscopy also insured we were in compliance of local treatment guidelines that required anyone positive by microscopy to receive prompt treatment.

Unless otherwise specified, malaria treatment was administered according to 2016 Cambodian National Treatment Guidelines. All volunteers with blood-stage parasitemia by microscopy were followed to clinical symptom resolution with two negative blood smears at least one week apart. If volunteers were only PCR positive, they were evaluated again the following month to ensure clearance. Recurrent cases of *Pf* were evaluated with molecular genotyping of MSP-1, MSP-2, and GLURP allelic variants to distinguish recrudescence from reinfection [30].

### Study interventions

During the intervention period, all participants in FSAT cohorts were screened monthly by RDT, microscopy and PCR (including at enrollment) and if positive for parasitemia by either microscopy or PCR, were treated following 2016 Cambodia National Treatment Guidelines, as follows. *Pf*-positive volunteers and those with mixed-species infections received a full three-day treatment course of artesunate and mefloquine (ASMQ, Guilin Pharmaceutical Co Ltd, Guilin, China) with a single low 15 mg PQ dose (Government Pharmaceutical Organization, Bangkok, Thailand). *Pv*-positive volunteers received a full three-day treatment course of DP plus 15 mg PQ daily for 14 days to prevent relapse. G6PD-deficient *Pv*-positive volunteers received 8 weekly 45 mg doses of PQ. Children < 13 years old were not treated with PQ. The intervention and follow-up period used the same intervention for FSAT subjects.

Volunteers in the MMP arm received three monthly three-day courses of fixed-dose combination DP tablets (Duo-cotexcin, Holley Pharmaceuticals, China) containing 40 mg of dihydroartemisinin and 320 mg of piperaquine phosphate during the intervention period. All MMP volunteers age ≥ 13 years also received 12 weekly fixed-dose PQ 22.5 mg tablets (270 mg total dose) to prevent malaria transmission and *Pv* relapse. MMP participants that were found to be malaria positive at enrollment received their first scheduled course of DP as prescribed in the MMP regimen instead of being given antimalarials based on the current standard-of-care defined by the national treatment guidelines. However, those MMP volunteers that were positive at enrollment and were still found to be positive during the first follow-up were then given a rescue treatment of standard-of-care antimalarials. Volunteers in the MMP arm who received rescue treatment continued their regularly scheduled MMP regimen with DP on next monthly follow up, and weekly low-dose PQ was continued on schedule without interruptions. The follow-up period for the MMP volunteers mirrored the FSAT intervention and follow-up period, reflective of the national treatment guidelines. All treatment for both MMP and FSAT cohorts was administered as DOT by the study team.

Participants were provided with civilian or military outer garments to wear in forested areas prior to study start as appropriate. Garments were pre-treated with insecticide by the study team prior to distribution using a single application of 40% permethrin Individual Dynamic Absorption kits (NSN 6840-01-345-0237; U.S. Army) to produce ITUs or water to produce sITUs. According to the product label, permethrin treatment was designed to withstand 30 washings.

### Statistical analysis

Detailed description of the statistical plan is outlined in the prior study protocol publication [29]. In brief, the primary study endpoint the absolute risk reduction (ARR) and relative risk reduction (RRR) based on the proportion of volunteers in each arm with PCR-corrected absence of parasitemia at the end of six months using an Intention-to-Treat analysis. Power analysis to determine the minimum sample size determined at least 78 individuals were required to achieve 80% power to detect a two-fold reduction in the ARR for MMP over FSAT with an α = 0.05 [29]. ARR was calculated separately for *Pf* and *Pv* and based on the proportion of volunteers that remained malaria free following enrollment out to six months [31]. Given that, by design, each treatment arm was balanced with respect to the number of cohorts with respect to uniform type and endemicity of their assigned location, we pooled the cohorts in each treatment arm to calculate an overall AAR and RRR for MMP vs. FSAT, with the rationale that if exposure level (whether due to uniform type or regional endemicity) was balanced between arms, then it should not have an impact on treatment efficacy. As the number of available cohorts was limited for practical reasons, a total of eight cohorts with 80 to 140 volunteers per cohorts was determined to be sufficient for this pilot study.

Demographic, epidemiological, and laboratory data were summarized at baseline and follow-up. All enrolled volunteers were included in the database for primary endpoint analysis. The safety analysis database included all volunteers who received at least one dose of study drug. ARR and RRR along with 95% confidence intervals and p-value are calculated as described in [32]. T-tests and chi-square tests were used to assess the statistical significance of differences in two means (including log-transformed) or proportions, respectively. Confidence limits (95%) for means, geometric means, and proportions were calculated as previously described [32]. A mixed-effects logistic regression model was used to assess the role of treatment arm (MMP vs. FSAT), insecticide-treated uniforms (ITU vs. sITU), regional endemicity (low vs. high), and parasitemia at enrollment (positive vs. negative) on the likelihood of a *Pf* or *Pv* infection during the 6 month study period. Random effects were modeled at the cohort level. Time to event data was summarized using Kaplan-Meier plots. The log-rank test was used to assess the statistical significance of differences between the treatment arms.

### Ethics statement

The study adhered to the CONSORT guidelines and the clinical trial was approved by the Walter Reed Army Institute of Research Institutional Review Board (WRAIR IRB, Protocol number, WR2211) and the National Ethics Committee for Health Research (NECHR) in Cambodia. The study was registered on Clinicaltrials.gov prior to enrollment (NCT02653898).

## Results

### Enrollment and study follow up

**Fig. 2:**
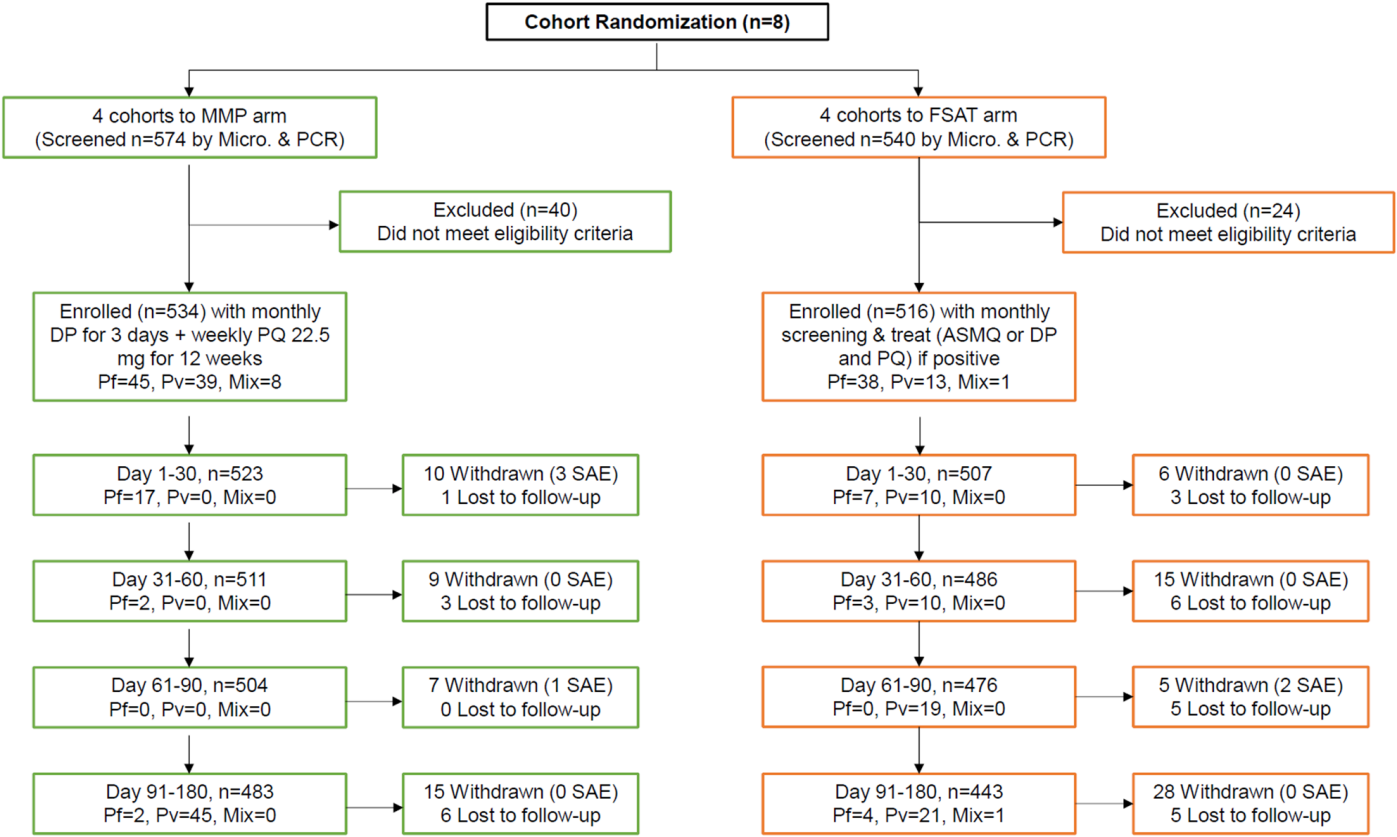
CONSORT Flow Diagram. Volunteers were randomized to one of 8 geographically isolated unit-based cohorts. There were a total of four cohorts for MMP (green) and another four cohorts for FSAT (orange) treatment interventions. Half of the volunteers assigned to MMP and FSAT were also treated with insecticide treated uniforms (ITUs). Total follow-up was 180 days after enrollment. Most volunteer withdrawals and losses to follow-up were due to movement outside the study areas

Of the 1,114 volunteers residing in the eight areas who were screened, 1,050 enrolled into the study (Fig. 2). This corresponded to 94.3% overall coverage in the population studied, ranging from 89 to 97% coverage within individual cohorts. The first subject was enrolled in January 2016 and the last subject follow-up was completed in August 2016. There were 534 volunteers in four cohorts assigned to the MMP arm (264 received ITU and 270 sITU) and 516 volunteers in four cohorts assigned to FSAT arm (240 received ITU and 276 sITU). Among enrolled volunteers, 75.8% (796/1050) were military personnel, 12.8% (134/1051) were non-military adults, and 11.4% (120/1050) were children < 18 years old. All volunteers identified ethnically as Khmer, and 84.3% (885/1050) of the population were male. At six months follow up, 90.1% (485/534) in MMP and 86.0% (443/516) in FSAT cohorts completed all study follow-up (Supplemental Table 1). Overall, only 11% (124/1050) withdrew from the study before completing all procedures, generally due to geographic reassignment or to new duties which precluded continuation of follow up.

Cohort composition at baseline is shown in Table 1. Baseline *Pv* prevalence was 8.4% (45/534) in the MMP groups compared to only 2.7% (13/516) in FSAT cohorts. *Pf* prevalence for both arms was comparable at baseline (*Pf* MMP = 62/534 = 9.7%; *Pf* FSAT = 42/516 = 8.1%). PCR-confirmed malaria prevalence at baseline was 15% (145/1050) across all cohorts, with *Pf* the predominant species at 8–10%. Overall, we found that cohorts in sites classified as high endemicity had an average baseline malaria positive rate of 18.8% while cohorts in sites classified as low endemicity had an average baseline positive rate of 8.0%, suggesting that the malaria incidence data used to classify the endemicity of these regions was reflected in the positivity rates at enrollment. The MMP cohorts had higher baseline malaria prevalence with 92 positive individuals consisting of 45 *Pf* cases (49%), 39 *Pv* cases (42%), and 8 mixed *Pf/Pv* infections (9%) by PCR. Of the 53 PCR-positive cases at baseline in the FSAT cohorts, there were 39 *Pf* cases (73%), 13 *Pv* cases (25%) and 1 mixed *Pf/Pv* infection (2%). Withdrawals and losses to follow-up were not evenly distributed among study cohorts, with a higher proportion withdrawing from the FSAT arm (14.5%), compared to the MMP arm (9.3%). Differences were nearly 2-fold higher after month 2 with 10.5% lost or withdrawn in the FSAT groups vs. 5.6% in MMP (Supplemental Table 1).

**Table 1:** Study cohort characteristics. Composition of 8 geographically isolated cohorts in the study including volunteer demographics, interventional assignments, baseline malaria prevalence, G6PD-deficiency prevalence and PQ treatments administered. Values are expressed as # (%) of those enrolled in each cohort in rows 6 through 15. The number screened represented the population of each cohort, with the number enrolled corresponding to intervention coverage (94% overall)

Cohort	A	B	C	D	E	F	G	H	Total
**Treatment Arm**	FSAT	MMP	MMP	FSAT	FSAT	FSAT	MMP	MMP	
**Uniform Assignment**	sITU	sITU	ITU	ITU	ITU	sITU	sITU	ITU	
**Regional Endemicity**	High	High	High	High	Low	Low	Low	Low	
**Number Screened**	151	150	134	129	121	139	140	150	1,114
**Number Enrolled**	143 (95)	142 (95)	130 (97)	125 (97)	115 (95)	133 (96)	128 (91)	134 (89)	1,050 (94)
**Non-military**	68 (48)	61 (43)	48 (37)	43 (34)	33 (29)	0 (0)	1 (1)	0 (0)	254 (24)
Children	32 (22)	32 (23)	33 (25)	13 (10)	10 (9)	0 (0)	0 (0)	0 (0)	120 (11)
Civilian adults	36 (25)	29 (20)	15 (12)	30 (24)	23 (20)	0 (0)	1 (1)	0 (0)	134 (13)
**Positive for Malaria on Screening by PCR**	29 (20)	29 (20)	28 (21)	17 (14)	4 (3)	3 (2)	22 (17)	13 (10)	145 (14)
*Pf* infection	23 (16)	17 (13)	12 (9)	12 (10)	2 (2)	2 (1)	11 (9)	5 (4)	84 (8)
*Pv* infection	5 (3)	11 (8)	11 (8)	5 (4)	2 (2)	1 (1)	9 (7)	8 (6)	53 (5)
Mixed *Pf/Pv* infection	1 (1)	1 (1)	5 (4)	0 (0)	0 (0)	0 (0)	2 (2)	0 (0)	9 (1)
**G6PD Deficiency** **WHO Class I-III**	15 (10)	17 (12)	25 (19)	15 (12)	13 (11)	25 (19)	23 (18)	27 (20)	160 (15)
G6PD Deficiency Primaquine-treated	5 (3)	16 (11)	25 (19)	2 (2)	2 (2)	1 (1)	22 (17)	24 (18)	97 (9.2)
> 10% Hemoglobin Drop at Day 3	0 (0)	3 (2)	6 (5)	0 (0)	1 (1)	0 (0)	3 (2)	1 (1)	14 (1.3)

### Treatment outcome by malaria species

**Fig. 3:**
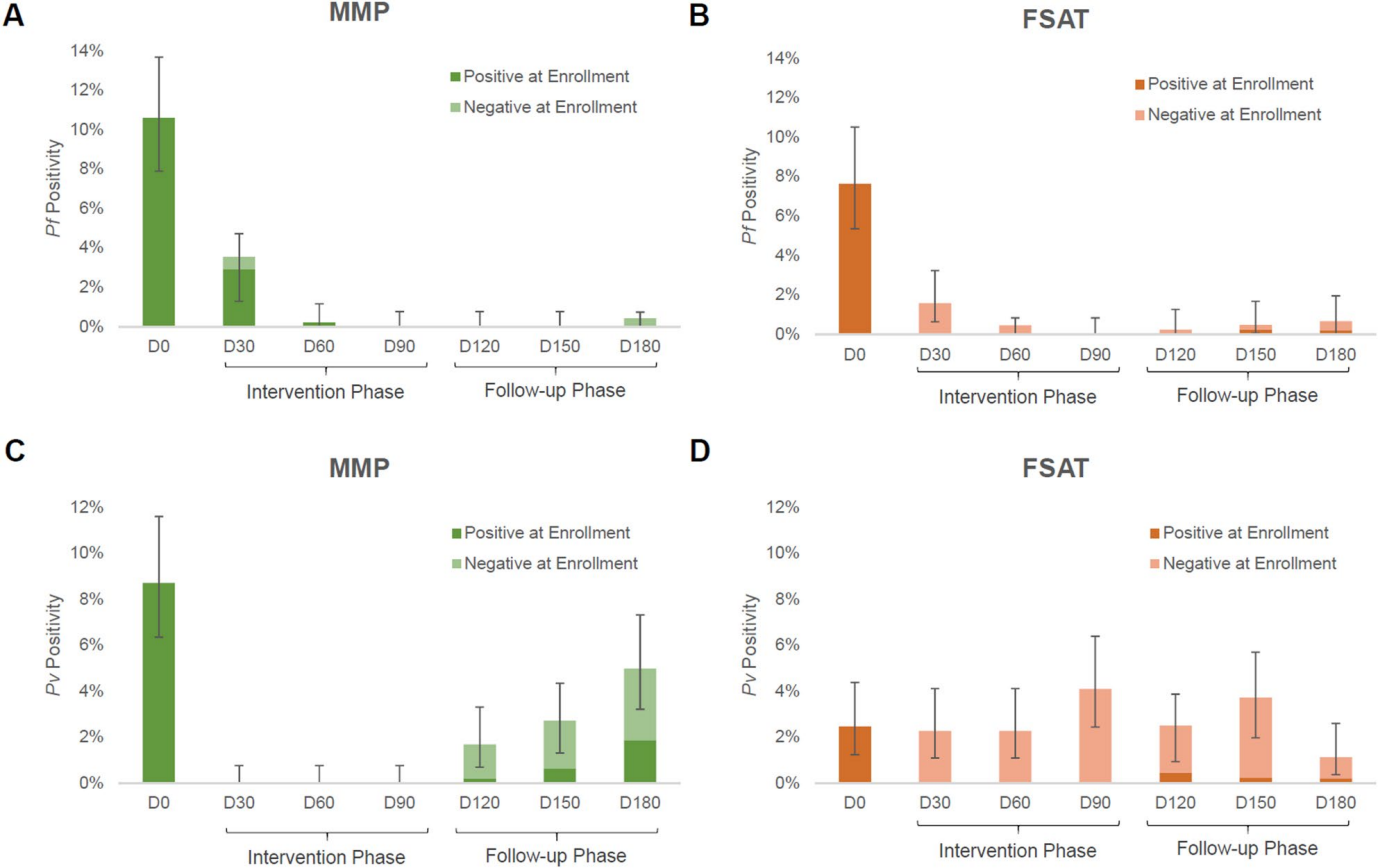
PCR-adjusted malaria prevalence on the monthly follow up in MMP and FSAT. Rapid and sustained reductions in the number of *Pf* malaria cases were observed in MMP (green) and FSAT (orange) (Panel **A**-**B**). A high rate of *Pv* malaria was detected for both interventions arms (Panel **C**-**D**) despite 270 mg total PQ dose in MMP arm (G6PD normal and G6PD deficient volunteers), over a 12 week period, and conventional doses of PQ in FSAT arm (i.e., 15 mg x 14 days in G6PD normal volunteers and 45 mg weekly x 8 weeks in G6PD deficient volunteers). Results are stratified between subjects that were positive at enrollment and tested positive at a follow-up time point and subjects that were negative at enrollment but tested positive at a follow-up time point. Error bars reflect the 95% confidence interval

**Fig. 4:**
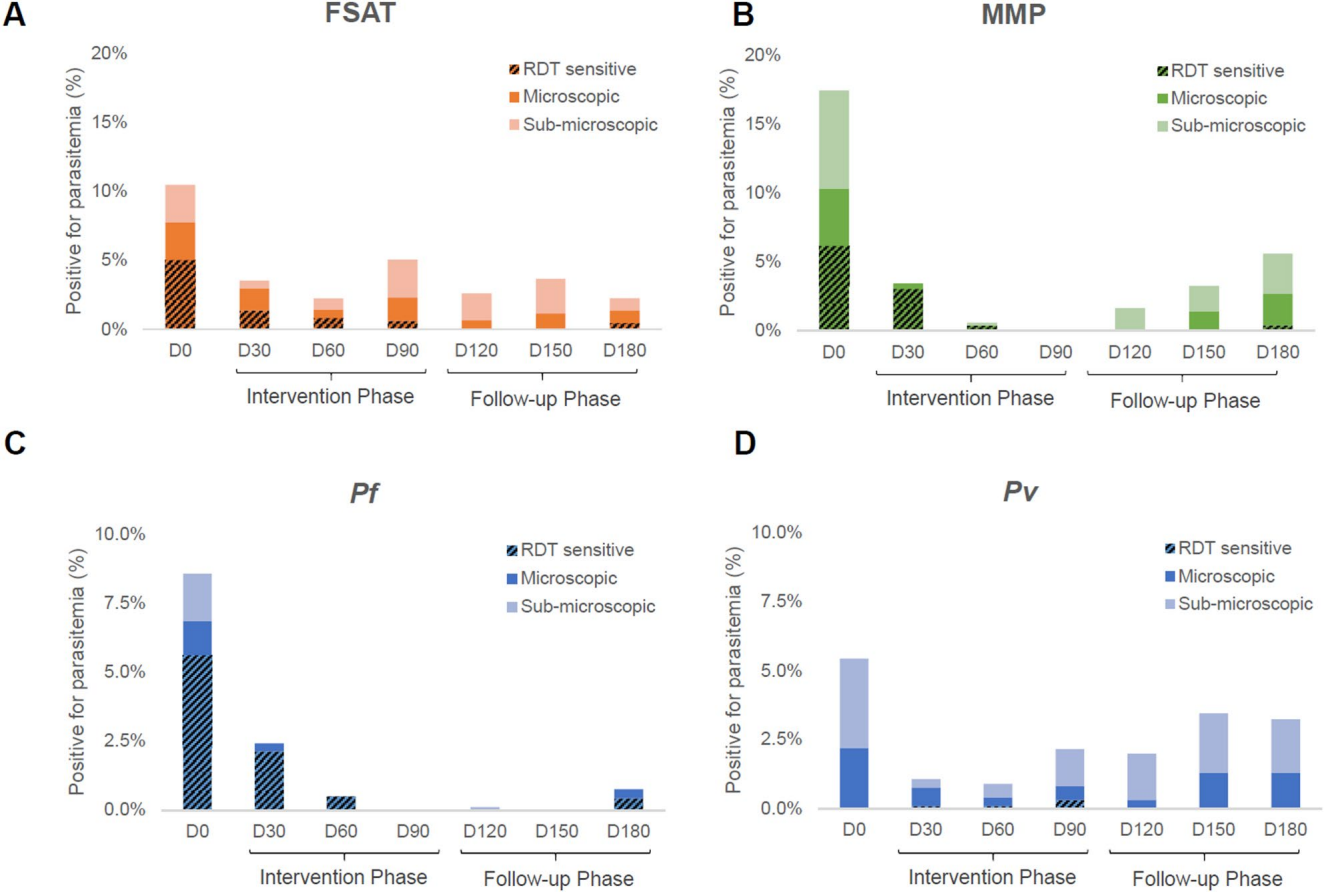
Diagnostic detection of malaria. Prevalence of all malaria species at each monthly follow up is shown based on the three diagnostic methods (RDT, microscopy, and PCR) performed on each individual at each visit. Participants were followed with parasitemia assessed monthly by rapid diagnostic test (RDT), microscopy (Micro) and plasmodium species-specific polymerase chain reaction (PCR) for 180 days. All RDT and Micro-positive cases were also PCR positive. Panels (**A**) and (**B**) compare detection rates between the (**A**) FSAT and (**B**) MMP arms. Panels **C**. and **D**. compare detection rates for all volunteers for (**C**) *P. falciparum* and (**D**) *P. vivax*

All cohorts experienced rapid *Pf* malaria reduction within 30 days of study initiation (Fig. 3A and B). At the last follow up on Day 180, less than < 1% of the study population had PCR-confirmed *Pf* malaria, with *Pv* being the predominant species at 2–4% (Figs. 2, 3 and 4). During the study period, based on the detected malaria cases, the predicted unadjusted prevalence of *Pf* was 11,048 episodes/100,000 population and the unadjusted prevalence of *Pv* was 15,238 episodes/100,000. From Month 1 to study end, 11.0% (95% CI: 8.0-11.7%) of volunteers experienced *Pv* infection and 15.4% (CI: 11.5–15.7%) of volunteers experienced *Pf* infection at least once.

*Pf* malaria transmission was interrupted by both MMP and FSAT interventions. PCR-corrected prevalence of *Pf* malaria declined from 9.9% at baseline to 0.4% on month 6 follow up (96% decline) for MMP and from 7.6% to 0.97% (87.1% decline) in FSAT treatment cohort (Fig. 3, **Panel A-B**). In MMP, 16 of 51 (31%) subjects that were positive for *Pf* at enrollment presented with malaria recurrence within their first two months of follow-up, with all 16 genotyped as recrudescences. All recurrent cases of *Pf* in the MMP cohort were successfully treated with ASMQ at the 1-month follow-up visit. In FSAT, where all *Pf*-positive volunteers were treated with ASMQ, 2/39 (5%) had PCR-confirmed recurrence within their first month of follow-up, and all cases were confirmed to be new infections. Volunteers assigned to the MMP arm experienced two cases of *Pf* in the last three months of follow-up, compared to four cases of *Pf* in FSAT arm, during the same follow-up period.

In contrast to *Pf* infections, ongoing *Pv* infections were observed in both treatment arms after enrollment during the study period. Despite rapid *Pv* decline in MMP, with no new cases detected during the treatment period (Days 0–90), 45 of 481 (9.3%) MMP subjects tested positive for *Pv* parasitemia after discontinuing prophylaxis within the 90-day follow-up period. In MMP subjects, 29% (13 of 45) of *Pv* cases during the follow-up period were in subjects that were positive for *Pv* at enrollment (Fig. 3, **Panel C**). In FSAT, overall *Pv* rates were twice as high as in MMP subjects (cumulative 81 of 444, 18.2%) as in Fig. 3, **Panel D**. *Pv* was more evenly distributed in the FSAT cohorts throughout the study period, with an average rate of 3.0% per month. *Pv* recurrences in individuals that were *Pv* positive at baseline made up a larger percentage of total *Pv* cases in the MMP arm compared to the FSAT arm (29% vs. 4.9%). The probability of *Pv* recurrence by 6 months if *Pv*-positive at baseline was similar for both arms (30% in MMP and 29% in FSAT). In contrast to MMP, only 4 of 81 *Pv* cases (4.9%) detected during follow-up in FSAT cohorts were in *Pv*-positive individuals at enrollment.

**Table 2:** Summary of absolute (ARR) and relative risk reductions (RRR) based on treatment arm

	Pf					
	D90			D180		
	Cases	Enrolled	Cumulative Pf rate	Cases	Enrolled	Cumulative Pf rate
FSAT Arm	9	472	1.9%	12	444	2.7%
MMP Arm	3	502	0.6%	5	481	1.0%
*ARR*	1.3% (-0.3%, 1.8%)			1.7% (-0.2%, 2.3%)		
*RRR*	68.7% (-15.0%, 91.5%)			61.5% (-8.5%, 86.3%)		
	*p* = 0.0804			*p* = 0.0711		

	Pv					
	D90			D180		
	Cases	Enrolled	Cumulative Pv rate	Cases	Enrolled	Cumulative Pv rate
FSAT Arm	40	472	8.5%	56	444	12.6%
MMP Arm	0	502	0.0%	32	481	6.7%
*ARR*	8.5% (6.9%, 8.5%)			6.0% (2.5%, 8.2%)		
*RRR*	100% (81.2%, 100%)			47.2% (20.1%, 65.2%)		
	*p* = 0.0017			*p* = 0.0025		

The primary endpoint of the study was the absolute and relative risk reduction (ARR and ARR, respectively) of the MMP arm compared with the FSAT arm (as a baseline) for *Pf* and *Pv* malaria at 6 months. A summary of ARR and RRRs are shown in Table 2, based on cumulative malaria positivity rates, defined as the total number of malaria cases identified from enrollment to Day 90 or Day 180. For cumulative *Pf* positivity, there was an RRR of 68.7% in MMP cohorts at day 90 relative to FSAT (0.6% vs. 1.9% prevalence) that decreased to an RRR of 61.5% at Day 180 (1.0% vs. 2.7% prevalence). For cumulative *Pv* positivity, the MMP arm showed a statistically significant RRR at both Day 90 and Day 180, with an RRR of 100% compared to FSAT (0% vs. 9.01%) at Day 90, that decreased to an RRR of 47% at Day 180 (12.6% vs. 6.7% prevalence). We also calculated the ARR and RRR at Day 180 for the ITU sub-arms relative to the sITU sub-arms as a baseline (Supplemental Table 2). For *Pf*, we saw RRRs of 61% and 100% for ITU sub-arm relative to sITU sub-arm within the FSAT, and MMP arms, respectively. For *Pv*, we found that sITU’s showed a statistically significant RRR of 57% in FSAT, but no significant RRR in the MMP arm. This discrepancy could be due to the fact that the MMP arm experienced a lower overall rate of *Pv* infections (6.7% vs. 12.6%) and a greater proportion of the Pv infections in this arm may have been due to relapses rather than new exposures (40% of new *Pv* infections in the MMP arm were in subjects positive for *Pv* at enrollment, compared to 6% in the FSAT arm).

For microscopy-positive cases, we did examine whether gametocytes could be observed to assess the potential effects of the intervention on transmission rates (Supplemental Fig. 1). We found that gametocytes were detected in a similar proportion of microscopy-positive cases in both the FSAT and MMP arms (50% vs. 39%, respectively). This suggests that the MMP arm did not have any additional effect on gametocyte formation, independent of its effect of overall infection rates.

### Diagnostic parasite detection

Participants were followed with parasitemia assessed monthly by RDT, PCR, and microscopy for 180 days to assess whether infections were RDT-sensitive and/or microscopic, or sub-microscopic (detectable by PCR only). Results are shown in Fig. 4 for the MMP and FSAT arms (**4 A-B**) and *Pf* and *Pv* for both arms (**4 C-D**). For the FSAT arm, we found that 17% of infections after Day 0 were RDT-sensitive, 51% were microscopic, and 49% were sub-microscopic. For MMP, we found similar results, with 26% of infections after Day 0 were RDT-sensitive, 54% were microscopic, and 45% were sub-microscopic. When separating by *Plasmodium* species we a pronounced difference in the presentation of *Pf* and *Pv* cases. For Pf cases, 70% were RDT-sensitive, 85% were microscopic, and 15% were sub-microscopic. By contrast, for *Pv* cases, only 3% were RDT-sensitive, 38% were microscopic, and 61% were sub-microscopic. In terms of assessment of the sensitivity and specificity of RDT and microscopy relative to PCR across the total of 6,784 tests conducted in this study, we found that microscopy was highly specific for both *Pf* (99.9%) and *Pv* (100%), but far more sensitive for *Pf* (84.3%) than *Pv* (39.0%). Microscopy was less sensitive than PCR, for both blood stage disease and gametocytes (3E-F). RDT was also highly specific for *Pf* (99.9%) and *Pv* (100%) compared to PCR but had poor sensitivity for *Pf* (69.4%) and virtually no sensitivity for *Pv* (0.5%).

### Effect of treated uniforms and endemicity on treatment outcome

**Fig. 5:**
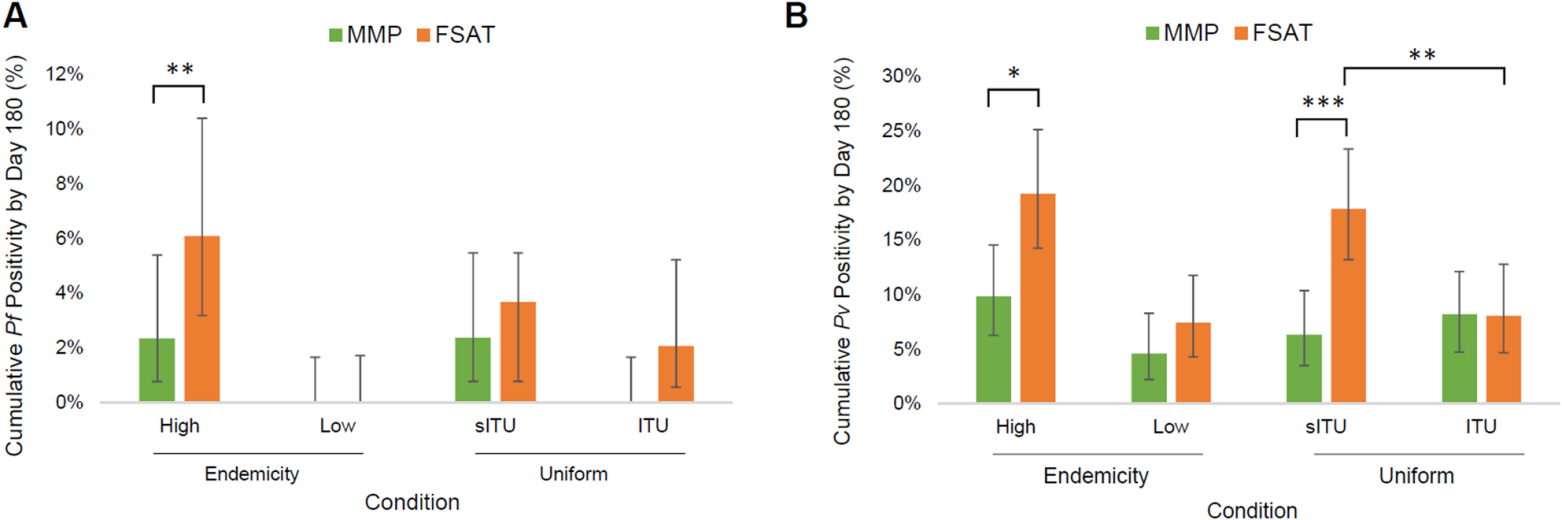
Cumulative *Pf* and *Pv* positivity with respect to drug intervention, uniform treatment, and endemicity. Cumulative positivity from Day 0 to 180 is shown for *Pf* (**A**) and *Pv* (**B**); MMP (green bars) and FSAT (orange bars) treatment conditions, stratified by cohort endemicity (high vs. low) and uniform (sITU vs. ITU). 95% confidence interval error bars and statistical significance (* *p* < 0.05, ** *p* < 0.01, *** *p* < 0.001) are shown

Mixed-effects logistic regression was used to assess effects of uniform treatment and endemicity on treatment outcome. Endemicity and uniform treatments are expected to affect the rate of acquiring *new* infections by affecting malaria vector exposure rates, but not rates of relapse or drug treatment failure. As such, this analysis included only subjects who were negative at enrollment for *Pf* or *Pv*, respectively, to assess the role of treatment and endemicity without confounding from treatment failure or relapse. Overall, for *Pf* infections, FSAT had approximately 3-fold higher cumulative *Pf* positivity among high-endemicity cohorts (*p* < 0.01) (Fig. 5A), whereas there were no new cases of *Pf* among subjects in the low-endemicity cohorts regardless of treatment arm. Although uniform treatment had no statistically significant effect of on cumulative *Pf* positivity, the overall total number of *Pf* cases in the study, particularly after Day 30 was low, limiting its statistical power. However, we note that cumulative *Pf* positivity in FSAT for those with sITUs was approximately double that of those with ITUs (3.9% vs. 1.9%), and that there were no new *Pf* cases in MMP subjects that were assigned ITUs (0%, compared with 2% for those assigned sITUs), suggesting that uniform treatment does have an effect on *Pf* infection rates.

There were significant effects of endemicity and uniform treatment on cumulative *Pv* positivity (Fig. 5B). Cumulative *Pv* positivity was approximately double in cohorts with high endemicity compared to cohorts with low endemicity. Stratifying by endemicity, FSAT subjects had approximately double the *Pv* positivity rate compared with MMP subjects in high endemicity cohorts (*p* < 0.05) but the effect was not statistically significant in the low endemicity cohorts. Stratifying by uniform type, FSAT subjects with sham-treated uniforms had almost 3-fold higher cumulative *Pv* positivity than MMP subjects with sham-treated uniforms (*p* < 0.001). However, there was no difference in *Pv* positivity between MMP and FSAT subjects wearing treated uniforms. FSAT subjects wearing sham-treated uniforms had almost 2.5-fold higher *Pv* positivity at 3 (6.3%) and 6 months (17%) than FSAT subjects wearing treated uniforms (2.2% and 7.3% respectively; RRR = 57.4% and ARR = 9.8% at 6 months; *p* < 0.01). See Supplemental Table 2 for summary logistic regression results.

### Malaria infection risk over time

Survival analysis was performed to evaluate risk of acquiring *Pf* and *Pv* infections over the course of the study. To ensure that the analysis was focused on new infections and not confounded by relapse or treatment failure, malaria positive subjects at enrollment were excluded. Time-to-event analysis was based on time to acquire the first documented *Pf* or *Pv* infection for each subject. The analysis was stratified by endemicity, and included only high endemicity cohorts for *Pf* as no new *Pf* infections were observed in low endemicity cohorts. *Pv* survival curves were stratified by uniform assignment (sITU vs. ITU) after logistic regression analysis showed endemicity and uniform were significantly associated with cumulative *Pv* positivity.

**Fig. 6:**
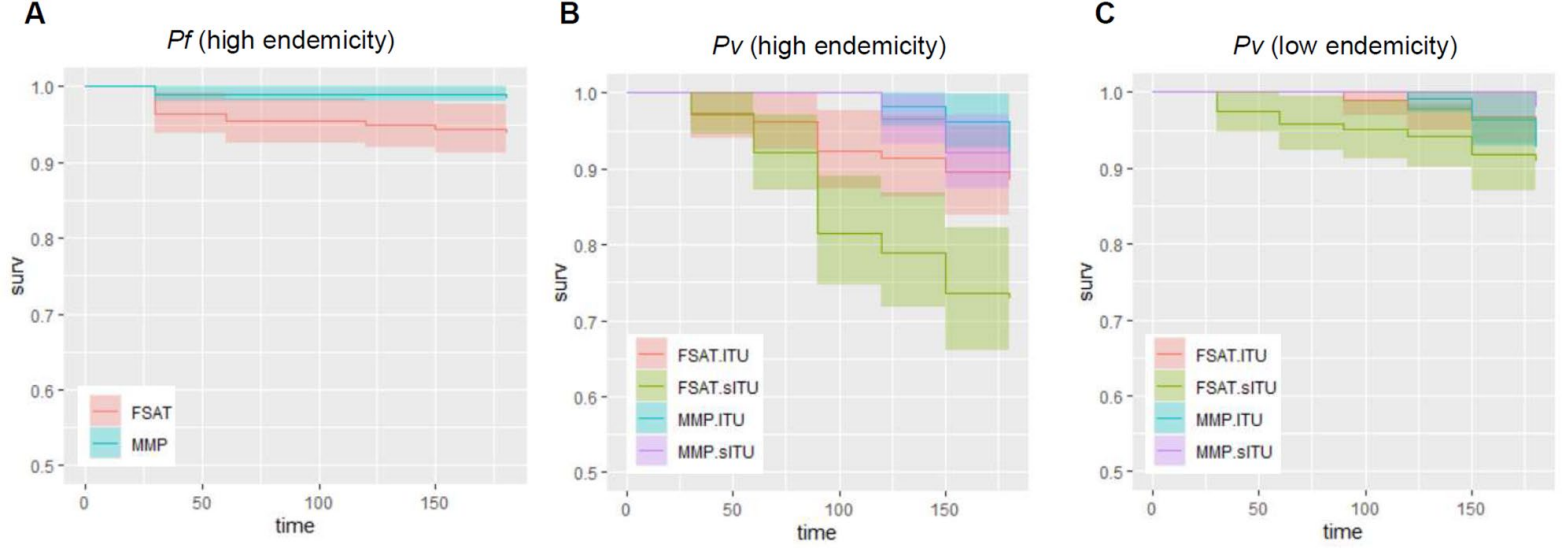
Survival curve to first *Pf* or *Pv* infection stratified by treatment. Survival curves are shown for *Pf* infection for FSAT (red) and MMP (blue) treatment conditions for in high endemicity cohorts (**A**). Survival curves for Pv infection in high and low endemicity cohorts (**B** and **C**) are shown for FSAT + ITU (red), FSAT + sITU (green), MMP + ITU (blue), and MMP + sITU (purple) conditions. 95% confidence intervals are shown as shaded colors corresponding to each condition. There was only 1 low endemicity *Pf* case which is not graphically displayed

Survival analysis showed significantly fewer *Pf* infections in the MMP than FSAT groups (Fig. 6A, *p* < 0.05), with no significant differences seen based on uniform treatment (not shown). For the *Pv* survival curve in the high endemicity cohorts, MMP treatment had significantly lower infection risk than FSAT treatment (*p* < 10^− 5^) and treated uniforms showed significantly lower infection risk than untreated uniforms (*p* < 0.05), with an interaction observed between treatment and uniform type (*p* < 0.05). Overall, in the high endemicity conditions, the FSAT + sITU condition performed significantly worse than the other three conditions (*p* < 10^− 7^). The *Pv* survival curve in low endemicity cohorts showed no statistically significant difference in infection risk for treatment or uniform type. However, there was a trend with higher infection risk for the FSAT + sITU condition than the other three. It is important to note, in the *Pv* survival curves, that while FSAT infection risk remained steady, the MMP infection risk was zero until D90, after which it increased to a level comparable to FSAT.

### Safety

Of 1,050 volunteers enrolled, there were 1,237 adverse events (AEs) reported over the 6-month study or 1.2 AEs per volunteer. The vast majority of AEs (88.0%) were classified as unlikely or not related to study interventions. Among the 162 study-related AEs, 98 were malaria infection, and 64 were study-related events other than malaria infection itself. Among FSAT cohorts, 13 subjects out of 516 (2.5%) experienced a study-related AEs besides malaria, of which two were of moderate severity (dizziness and hemolysis, in both cases, within one week of being treated for *Pv*). Among MMP cohorts, 42 subjects out of 534 (7.8%) experienced a study-related AE besides malaria, including five of which were moderate (all hemolysis) and one of which was severe (migraine headache). The most common study-related AEs in the MMP cohorts were hemolysis (8 mild events, 5 moderate events), stomach upset (12 mild events), and gastritis (8 mild events). Overall, 98.9% of subjects in the MMP cohorts and 99.6% of subjects in the FSAT cohorts experienced either no study-related AEs or mild study-related AEs, besides malaria infection.

There were 27 SAEs (2.6%) recorded during the study period and are listed in Supplemental Table 4. All were determined to be unrelated to the study interventions except for one that was classified as ‘unlikely related’ (bronchopneumonia with subacute pulmonary edema) and one that was ‘possibly’ related (new onset migraine headache which resulted in inpatient treatment). Of the 25 SAEs unrelated to the study, eight were due to traumatic injuries associated with traffic accidents, six were attributed to causes related to alcohol or substance abuse, and eight were due to a variety of conditions include pneumonia, cellulitis, enterocolitis, or appendicitis. All volunteers with SAEs were followed until recovery or until their condition has stabilized. One volunteer was diagnosed with breast cancer and was withdrawn from the study while receiving treatment. One volunteer was withdrawn due to intolerance to DP in MMP arm (vomiting). One volunteer died from causes unrelated to study interventions with causality assessed by the research monitor, an independent review, and the ethics review board.

While there have been reports of clinically significant hemolysis observed following PQ treatment even in G6PD normal individuals [33], no significant hemolysis occurred in G6PD-deficient volunteers taking primaquine during the study. All volunteers with moderate to severe G6PD deficiency tolerated the modified 12 weekly doses of PQ without clinically significant hemolysis. There were 160 volunteers (15%) with G6PD-deficiency identified at screening. Of these, 97 (9.2%) took primaquine during the study, but only 14 (1.3%) had a hemoglobin decline >10% at day 3 (Table 1). No volunteers had a hemoglobin drop >25% from baseline or required stopping therapy.

## Discussion

This study was designed to evaluate MMP and FSAT approaches to address four aims of effective malaria treatment and elimination: (1) clearing blood stage infection; (2) reducing transmission risk; (3) clearing latent liver-stage infection (for *Pv*); and (4) lower the risk of reinfection by reduction of malaria burden. Insecticide-treated clothing was assessed for added benefit against day-biting mosquitoes in endemic forested areas.

In the MMP regimen, these four therapeutic objectives above were achieved using DP to treat acute parasitemia and provide suppressive chemoprophylaxis (Objectives 1 and 4), and low-dose weekly PQ to reduce transmission risk, eliminate or suppress latent *Pv* infection (Objective 3) and provide causal liver-stage chemoprophylaxis (Objectives 2, 3, and 4). MMP cohorts reached zero PCR-detectable malaria parasitemia after 3 months of treatment. There was no microscopic gametocytemia detected between Months 2–6 or blood-stage *Pv* by PCR between Months 2–4. There were relative risk reductions (RRR) of 60–70% in cumulative *Pf* infection in the MMP arm compared to FSAT over the course of the study. The RRR of MMP relative to FSAT was 100% for *Pv* after the three-month intervention period, and 47% through 6 months of follow-up. Uptake of therapy was excellent, well-tolerated, and compliance was high. The primary drawback for MMP in this study was the high baseline *Pf* resistance to DP in Cambodia. The ~ 30% clinical failures in the MMP arm required rescue therapy with ASMQ at Month 1, all of which were subsequently cured and remained *Pf* negative thereafter.

MMP findings were in contrast to those among the FSAT cohorts, particularly for *Pv* where *Pv* parasitemia among the FSAT cohorts remained endemic at a rate of 2–4% throughout the study. During the follow-up period when MMP and FSAT arms had the same screening/treatment regimen, malaria re-emerged in the MMP arm, with *Pv* parasitemia detected within one month and *Pf* within three months after the intervention period. Although the study was not powered to assess the effectiveness of insecticide-treated clothing, there was a statistically significant benefit against *Pv* infection in the FSAT but not MMP arm, suggesting that uniform treatment may provide additional protection from malaria absent chemoprophylaxis as part of a screen-and-treat approach.

The appropriate selection of diagnostics methods is critical for malaria elimination efforts. In this study, PCR was the definitive diagnostic method for case definition because of its high sensitivity. We found that for *Pf*, RDT and microscopy showed high sensitivity, with 70% of cases being detectable by RDT and 85% detectable by microscopy. By contrast, for *Pv*, only 3% of cases were RDT-sensitive, and only 38% were microscopic, with 62% of cases being detectable by PCR only. This highlights the challenges of relying on non-molecular methods such as RDT or microscopy for *Pv* screening and underscores the advantage of an MDA approach that does not rely on costly molecular screening methods that are challenging to implement in resource-limited settings. We found that the proportion of cases that was RDT-sensitive, microscopic, and sub-microscopic was not significantly different between the FSAT and MMP arms, suggesting that treatment type does not significantly impact the infection presentation (microscopic vs. sub-microscopic) of resulting malaria cases – a concern in some malaria elimination settings.

A recent systematic review identified at least 10 randomized trials evaluating malaria MDA in [16] and found that both *Pf* and *Pv* burden were substantially reduced within 1–3 months in low transmission settings. A prior systematic review of 32 studies found that MDA led to sustained malaria reductions only in geographically isolated communities [17, 32]. One key lesson from our study is the need for sustained intervention beyond three months. Although impact is often relatively short-lived, MDA approaches to malaria elimination appear to be more effective in low intensity transmission settings such as Cambodia (1–10% prevalence). The most challenging elements to account in any MDA study are movements in and out of cohorts and the cohorts on the Cambodian-Thai border in our study were not truly geographically isolated from neighboring malaria risk areas. Despite these challenges, the present study sustained parasite-free intervals in MMP cohorts longer than most prior efforts with five months before PCR-confirmed *Pf* reappeared in the study.

Cambodia has long been among the highest risk countries for antimalarial drug resistance for both *Pf* [34] and *Pv* [35] and high rates of drug failure and drug tolerability issues at the time limited possible elimination regimens available for this study [3, 36]. Given a lack of treatment options when the study was implemented, a monthly three-day DP course was chosen as the preferred chemoprophylaxis in the MMP arm despite clinical DP treatment failures widely reported in the study area three years before study start [37]. At the time of the study, Cambodia had recently replaced DP with ASMQ as first line *Pf* agent with a single 15 mg dose of PQ to prevent transmission based on findings of inverse resistance patterns observed between DP and ASMQ in Northern Cambodia [34]. In line with these findings, we observed a 32% initial DP failure rate in the MMP arm in the first month, compared to no ASMQ regimen failures in the FSAT arm, and only 3 ASMQ recrudescences over the remaining 6 months. All treatment failures in the MMP arm were successfully cured using ASMQ rescue therapy on month 1 follow-up. Despite the initial DP treatment failures, DP chemoprophylaxis in the MMP arm significantly outperformed FSAT using ASMQ treatment in terms of cumulative *Pf* infection rates. Our study was not alone in selecting DP as a monthly blood stage elimination agent. The closest recent comparable study used monthly courses of DP with single low-dose PQ to eliminate *Pf* in Vietnam, Cambodia, Laos and Myanmar [38] and successfully achieved *Pf* suppression for as long as one year in lower transmission areas. We did not find any significant drug resistance at the study sites and observed that 9 of 10 cases of highly resistant *PfPailin* strain were cured with initial therapy.

Presently, *Pv* is the greatest long-term obstacle to malaria elimination in Cambodia and SE Asia [39]. *Pv* constituted 57% of overall malaria and 88% of malaria detected after initial enrollment in the present study. Here, MMP effectively suppressed or eliminated blood-stage *Pv* during the three-month intervention period with zero detected cases of *Pv* through Month 3. By contrast, monthly blood-stage *Pv* rates in the FSAT arm remained largely unchanged between 2 and 4% throughout the study period. However, interpretation of these results is confounded by the complexity of distinguishing relapses, recrudescences, and reinfections [40]. In this study, it was impossible to determine whether MMP was effective against *Pv* because it cleared hypnozoites, interrupted transmission, reduced reinfection, or simply suppressed apparent blood-stage relapses. The results based on the timing of infection suggest that MMP *Pv* recurrences were proportionately more likely to be the result of relapses, while FSAT recurrences are proportionately more likely to be the result of reinfections (Supplemental Fig. 2). The beneficial effect of ITU on *Pv* rates in the FSAT arm further supports the notion that reinfections played a significant role in FSAT *Pv* rates. The difference in intervals between the two regimens may be due to a combination of factors – a higher reinfection rate of new *Pv* infections in FSAT, suppression of detectable relapse during the intervention period in MMP, or both. However, it is clear that the FSAT approach had little effect on *Pv* rates despite prompt administration of PQ radical cure following detection.

A fixed 22.5 mg weekly PQ dose was previously found to be effective in eliminating latent *Pv* in immigrants to Australia, and was the basis of the MMP regimen here [41]. In this study, that dose was selected as a ‘universal’ G6PD-safe dose that could be safely used in a mass drug administration context where individualized dosing [42], G6PD testing, and routine monitoring for risks like hemolysis may not be feasible. Whether this low dose of PQ would be sufficient to prevent relapse was not clear at the onset of this study. We found that *Pv* at enrollment was the single greatest predictor of *Pv* positivity during the study using logistic regression, suggesting that relapse, in fact, likely played a major role in *Pv* infection rate in this study. In both MMP and FSAT arms, approximately 30% of individuals that were positive for *Pv* at enrollment and treated with PQ under their respective regimens had a *Pv* recurrence during the study. This compared to an overall cumulative *Pv* infection rate of 7.4% in MMP and 15.6% in FSAT for subjects that were *Pv* negative at enrollment. These findings suggest that regardless of the different PQ regimens used in MMP and FSAT, the cumulative 270 mg dose of PQ (in the MMP arm) and 210 mg dose of PQ (in the FSAT arm) may not be completely effective at preventing relapse. This is in line with a recent study that found that PQ radical cure may only be 60–70% effective at preventing recurrences in this setting [43]. It is important to note that the current WHO treatment standard for G6PD deficient patients is 0.75 mg/kg for 8 weeks, which for a 60 kg adult is equivalent a 360 mg total dose [44], significantly higher than the 270 mg dose used in the MMP arm.

Malaria elimination efforts are unlikely to be successful without suitable attention paid to the infectious gametocyte. Treatment of blood stage parasites can stimulate gametocytemia and increase transmission risk [45]. Prior studies have shown that PQ is the most effective drug for treating stage V gametocytes in the human host to block mosquito transmission [46]. A single 45 mg dose of PQ administered with blood-stage therapy was shown to be a highly effective transmission-blocking agent in a similar geographical areas as the present study [47], eliminating circulating *Pf* gametocytes and preventing transmission from patient blood samples to laboratory reared mosquitoes. A lower single 15 mg dose is now widely recommended for transmission-blocking in combination with a blood stage agent. While the MMP and FSAT arms were treated with PQ, the MMP arm received a weekly dose of PQ well in excess of this recommended transmission-blocking dose. The FSAT arm received a single transmission-blocking dose only if confirmed *Pf* positive or a radical curative dose only if *Pv* positive, leaving subclinical gametocytemia untreated. In this study, microscopic gametocytemia was measured, which is thought to capture the bulk of transmission risk from blood-stage parasitemia [48]. In the MMP arm, no microscopic *Pv* gametocytes were observed from Months 1–4 and no *Pf* gametocytes from Months 1 to 6. In the FSAT arm, while no *Pf* gametocytes were observed from Months 2–6, *Pv* gametocytes were seen in 50–67% of FSAT *Pv* infections throughout the entire 6-month observation period, suggesting ongoing *Pv* transmission throughout the study. This may help explain why the addition of ITU was shown to be beneficial in FSAT where transmission was never interrupted with the drug therapies alone.

The present study demonstrated high compliance, safety, and tolerability through the use of DOT administration. DP is known to be safe and well-tolerated with the exception of elevated risk of QT-interval prolongation when given in a compressed 2-day course [49]. The primary safety concern in the study was use of PQ in G6PD-deficient volunteers. We were the first group to employ a universal 22.5 mg/week PQ dose regimen in SE Asia with the intent that such a regimen could be used safely without the need for G6PD testing or individualized dosing. In the MMP arm, 16% of volunteers were G6PD deficient, comparable to prior observations [50]. Only 15% of subjects had hemoglobin declines of >10% at day 3, none had declines of >25%, and none had to stop PQ treatment based on the pre-specified individual halting rule of Grade 3 hemolysis. Overall, using the fixed 22.5 mg weekly dose, 97.5% of MMP subjects reported no hemolysis adverse events, 1.4% reported mild hemolysis events, and 0.9% reported moderate hemolysis events, with no reports of severe hemolysis. This is in line with expectations that even those with relatively severe G6PD deficiency are able to tolerate PQ in low doses [51], suggesting that ‘universal’ PQ was well tolerated regardless of G6PD status or individual variations in body size.

One key feature of the present study was assessing the effectiveness of pharmaceutical approaches (MMP and FSAT) to malaria elimination in combination with a vector-based intervention. Insecticide-treated clothing, typically using wash-resistant permethrin formulations, is often employed by military forces to reduce vector-borne disease risk. The vast majority of vector-based interventions are tested based on their efficacy against vector biting behavior and few are evaluated within the context of a clinical trial [27]. We found that in the FSAT arm, ITUs resulted in significantly lower *Pv* positivity rates (*p* < 0.01) with RRR of 58% for *Pv* compared to sITU, with a similar effect size seen for *Pf* with RRR of 61% suggesting that ITUs can be effective in regions with day-biting vectors. It is interesting to note similar effect sizes for both *Pf* and *Pv*, which is consistent with both species sharing the same vector. No statistically significant difference was found in *Pf* and *Pv* infection rates for ITUs vs. sITUs in the MMP arm, suggesting that the chemoprophylactic effects of the MMP regimen may have masked any beneficial effects of uniform treatment.

There were several limitations to the study. First, despite randomization, the multiplicity of simultaneous interventions makes it difficult to quantify relative contributions of individual interventions. This was the result of a deliberate choice. The goal of the study was to combine the most regionally appropriate, safe, cost-effective elimination strategies available at the time rather than precisely delineate individual contributions of each intervention. Second, assessment of potential reinfection for *Pv* was challenging, given the difficulties in distinguishing relapse, reinfection and recrudescence. Third, we could not control for the effects of in- and out-migration. The former may have introduced additional disease burden to cohorts while the latter caused losses to follow-up from occupational reassignment. Fourth, secular declines in parasite prevalence from either seasonal changes or ongoing control efforts in the region were also impossible to quantify. A no-intervention arm may have helped control for this, but were not undertaken for both ethical and practical considerations. To our knowledge, study sites selected did not undergo other control measures beyond those of the study during the observation period. Also, the study concluded during a time of the year generally considered to be a higher malaria transmission period. The enrollment began during low malaria transmission (January) and concluded during the rainy season (June) when malaria transmission in Cambodia typically increases [52]. Seasonality would likely have contributed to a secular rise, rather than fall, in infection rates over the course of the study if the control measures were ineffective. Finally, selection bias may have influenced cohort composition, though unavoidable for practical reasons. This was mitigated by site selection based on several years of prior malaria data provided by the RCAF.

## Conclusions

The malaria elimination study described here among mobile populations on the Cambodian-Thai border demonstrated that a chemoprophylaxis strategy (MMP) was more effective than a reactive-screen-and-treat approach (FSAT). The effects of MMP were longer lasting after the intervention was completed. This was true for both *Pf* and *Pv*, though in this area of MDR *Pf*, follow-up surveillance and retreatment were necessary for 30% of volunteers treated initially with DP. A weekly low dose of 22.5 mg primaquine was safe, well-tolerated, and appeared to potentiate DP prophylactic effects particularly against *Pv*. FSAT was not effective in reducing *Pv* prevalence. Insecticide-treated clothing appeared to provide additional benefit with the FSAT but not MMP approach. The MMP approach may be more easily scaled to eliminate malaria given reduced need for diagnostic assessments. The study informs a number of outstanding malaria elimination questions, and highlights regional militaries as a potent force for malaria elimination in the Southeast Asia.

## Supplementary Information

Below is the link to the electronic supplementary material.

Supplementary Material 1

Supplementary Material 2

## Data Availability

The dataset supporting the conclusions of this article is included within the article and its additional files.

## Declarations

### Disclaimer

The views expressed in this article are those of the authors and do not reflect the official policy of the US Department of the Army, US Department of Defense, the US Government or the Royal Government of Cambodia.
