# Supplementary material for: Evaluating malaria elimination strategies among military forces in Cambodia: a multi-arm clinical trial comparing monthly prophylaxis and focused screening and treatment
Source: BMC Infect Dis. 2025 Dec 12;26:84. doi: 10.1186/s12879-025-12207-4 (PMC12817837; doi:10.1186/s12879-025-12207-4)
Supplement: Supplementary file 2 — Supplementary Material 2 [file 12879_2025_12207_MOESM2_ESM.docx]

# Supplemental Material

**Supplemental Table 1.** Withdrawals (W) and losses to follow-up (LTF) over time by treatment arm and total volunteers (TOT).

|  | ***Pf*** | | |  | ***Pv*** | | |
| --- | --- | --- | --- | --- | --- | --- | --- |
|  | **D180** | | |  | **D180** | | |
|  | **Cases** | **Enrolled** | **Cumulative *Pf* rate** |  | **Cases** | **Enrolled** | **Cumulative *Pv* rate** |
| FSAT.sITU | 9 | 239 | 3.8% |  | 41 | 239 | 17.2% |
| FSAT.ITU | 3 | 205 | 1.5% |  | 15 | 205 | 7.3% |
| *ARR* | 2.3% (-1.6%, 3.4%) | | |  | 9.8% (-4.3%, 13.0%) | | |
| *RRR* | 61.1% (-41.6%, 89.4%) | | |  | 57.3% (24.9%, 75.6%) | | |
|  | p = 0.1520 | | |  | p = 0.0031 | | |
|  |  |  |  |  |  |  |  |
| MMP.sITU | 5 | 239 | 2.1% |  | 12 | 239 | 5.0% |
| MMP.ITU | 0 | 242 | 0.0% |  | 20 | 242 | 8.3% |
| *ARR* | 2.1% (-1.3%, 2.1%) | | |  | 3.6% (1.4%, 4.7%) | | |
| *RRR* | 100% (-61.4%, 100%) | | |  | -64.6% (-129%, 17.8%) | | |
|  | p = 0.1021 | | |  | p = 0.1587 | | |

**Supplemental Table 2.** Absolute Risk Reduction (ARR) and Relative Risk Reduction (RRR) for the ITU sub-arms relative to the sITU sub-arms in the FSAT and MMP treatment arms.

| ***Pf - high endemicity*** | |  |  | **95% C.I.** | |  | **95% C.I.** | |  |
| --- | --- | --- | --- | --- | --- | --- | --- | --- | --- |
|  | **Total** | ***Pf* Positive** | **Percent** | **lower** | **upper** | **Odds-Ratio** | **lower** | **upper** | **p-value** |
| FSAT.sITU | 97 | 8 | 8.2% | 3.6% | 15.6% | *Reference* |  |  |  |
| FSAT.ITU | 105 | 4 | 3.8% | 1.1% | 9.5% | 0.464 | 0.135 | 1.593 | n.s. |
| MMP.sITU | 107 | 5 | 4.7% | 1.5% | 10.6% | 0.545 | 0.172 | 1.727 | n.s. |
| MMP.ITU | 106 | 0 | 0.0% | 0.0% | 3.4% | - | - | - | - |
|  |  |  |  |  |  |  |  |  |  |
| ***Pv - high endemicity*** | |  |  | **95% C.I.** | |  | **95% C.I.** | |  |
|  | **Total** | ***Pv* Positive** | **Percent** | **lower** | **upper** | **Odds-Ratio** | **lower** | **upper** | **p-value** |
| FSAT.sITU | 113 | 30 | 26.5% | 18.7% | 35.7% | *Reference* |  |  |  |
| FSAT.ITU | 105 | 12 | 11.4% | 6.1% | 19.1% | 0.357 | 0.172 | 0.742 | 0.0056 |
| MMP.sITU | 115 | 12 | 10.4% | 5.5% | 17.5% | 0.322 | 0.155 | 0.668 | 0.0023 |
| MMP.ITU | 108 | 10 | 9.3% | 4.5% | 16.4% | 0.282 | 0.13 | 0.612 | 0.0013 |
|  |  |  |  |  |  |  |  |  |  |
| ***Pv - low endemicity*** | |  |  | **95% C.I.** | |  | **95% C.I.** | |  |
|  | **Total** | ***Pv* Positive** | **Percent** | **lower** | **upper** | **Odds-Ratio** | **lower** | **upper** | **p-value** |
| FSAT.sITU | 122 | 12 | 9.8% | 5.2% | 16.6% | *Reference* |  |  |  |
| FSAT.ITU | 93 | 4 | 4.3% | 1.2% | 10.7% | 0.444 | 0.137 | 1.44 | n.s. |
| MMP.sITU | 106 | 2 | 1.9% | 0.2% | 6.7% | 0.192 | 0.042 | 0.889 | n.s. |
| MMP.ITU | 111 | 8 | 7.2% | 3.2% | 13.7% | 0.777 | 0.301 | 2.01 | n.s. |

**Supplemental Table 3. Summary logistic regression analysis results.** All comparisons are made with respect to FSAT randomized to sham-treated uniforms (FSAT+sITU). In high Pf-endemicity clusters, both FSAT with treated uniforms and MMP with untreated uniforms had 2-fold less risk for malaria infection (ns). In high Pv endemicity clusters, FSAT with treated uniforms or MMP with or without treated uniforms had a roughly 3-fold significant decrease (95% CI of 1.5-fold to 6.5-fold) in malaria risk (p < 0.01). There was a trend toward decrease in Pv risk in low endemicity clusters associated with treated uniforms or MMP (ns).

**Supplemental Table 4.** Summary of Severe Adverse events in the study

| **No** | **Subject ID** | **Event/Diagnosis** | **Start date** | **End date** | **Relationship to Study drugs** |
| --- | --- | --- | --- | --- | --- |
| 1 | GF2211B-011 | Laceration wounds on the forehead due to a traffic accident | 24 Jan 2016 | 01 Feb 2016 | Not related |
| 2 | GF2211B-027 | Severe headache | 31 Jan 2016 | 04 Feb 2016 | Possibly |
| 3 | GF2211D-054 | Ulcerative gastritis with melena | 05 Feb 2016 | 14 Feb 2016 | Not related |
| 4 | GF2211B-138 | Multiple laceration wounds on the forehead and face due to a traffic accident | 07 Feb 2016 | 15 Feb 2016 | Not related |
| 5 | GF2211C-085 | Pneumonia | 10 Feb 2016 | 15 Feb 2016 | Not related |
| 6 | GF2211C-117 | Bronchopneumonia, three-fourths mitral and one-fourth aortic insufficiency, with subacute pulmonary edema | 12 Feb 2016 | 19 Feb 2016 | Unlikely |
| 7 | GF2211H-046 | Alcoholism with co-complicated symptoms | 22 Feb 2016 | 25 Feb 2016 (Dead) | Not related |
| 8 | GF2211A-056 | Symptoms of mild intoxication post ingestion of insecticide lotion | 27 Feb 2016 | 28 Feb 2016 | Not related |
| 9 | GF2211G-036 | Right lower extremity cellulitis | 03 Mar 2016 | 09 Mar 2016 | Not related |
| 10 | GF2211D-005 | Left foot cellulitis | 08 Mar 2016 | 14 Mar 2016 | Not related |
| 11 | GF2211D-057 | Closed fracture of 1/3 right clavicle and trauma of right thorax | 09 Mar 2016 | 16 Mar 2016 | Not related |
| 12 | GF2211E-041 | Acquired pneumonia | 16 Mar 2016 | 20 Mar 2016 | Not related |
| 13 | GF2211F-027 | Dengue fever | 21 Mar 2016 | 26 Mar 2016 | Not related |
| 14 | GF2211E-040 | Chronic Alcoholism with complications, psychosis | 27 Mar 2016 | 29 Mar 2016 | Not related |
| 15 | GF2211H-100 | Enterocolitis | 29 Mar 2016 | 01 Apr 2016 | Not related |
| 16 | GF2211E-041 | Alcoholism with neuralgia and polyneuritis | 01 Apr 2016 | 12 Apr 2016 | Not related |
| 17 | GF2211G-040 | Epilepsy and alcohol intoxication withdrawal | 04 Apr 2016 | 06 Apr 2016 | Not related |
| 18 | GF2211E-028 | Laceration wound above right eyebrow due to a traffic accident | 12 Apr 2016 | 19 Jun 2016 | Not related |
| 19 | GF2211D-004 | Appendicitis | 26 Apr 2016 | 12 May 2016 | Not related |
| 20 | GF2211E-039 | Suspected alcohol withdrawal syndrome with dysentery | 08 May 2016 | 09 May 2016 | Not related |
| 21 | GF2211D-122 | Lacerated wound on left upper eye brow due to motorcycle accident | 7 May 2016 | 11 May 2016 | Not related |
| 22 | GF2211H-026 | Lacerated wound on left forearm and foot due to motorcycle accident | 17 May 2016 | 22 May 2016 | Not related |
| 23 | GF2211H-128 | Left tibia fracture, facial skin scratch and left sole injury due to motorcycle accident | 19 May 2016 | 27 May 2016 | Not related |
| 24 | GF2211E-039 | 2^nd^ degree skin burn on left side body due to epilepsy | 26-Jun-16 | 11 July 2016 | Not related |
| 25 | GF2211E-115 | Pneumonia | 01 Jul 2016 | 05 Jul 2016 | Not related |
| 26 | GF2211F-046 | Prostatic urethra stone | 11 Jul 2016 | 17 Jul 2016 | Not related |
| 27 | GF2211C-095 | Right breast cancer | 15 July 2016 | 12 Aug 2016 | Not related |


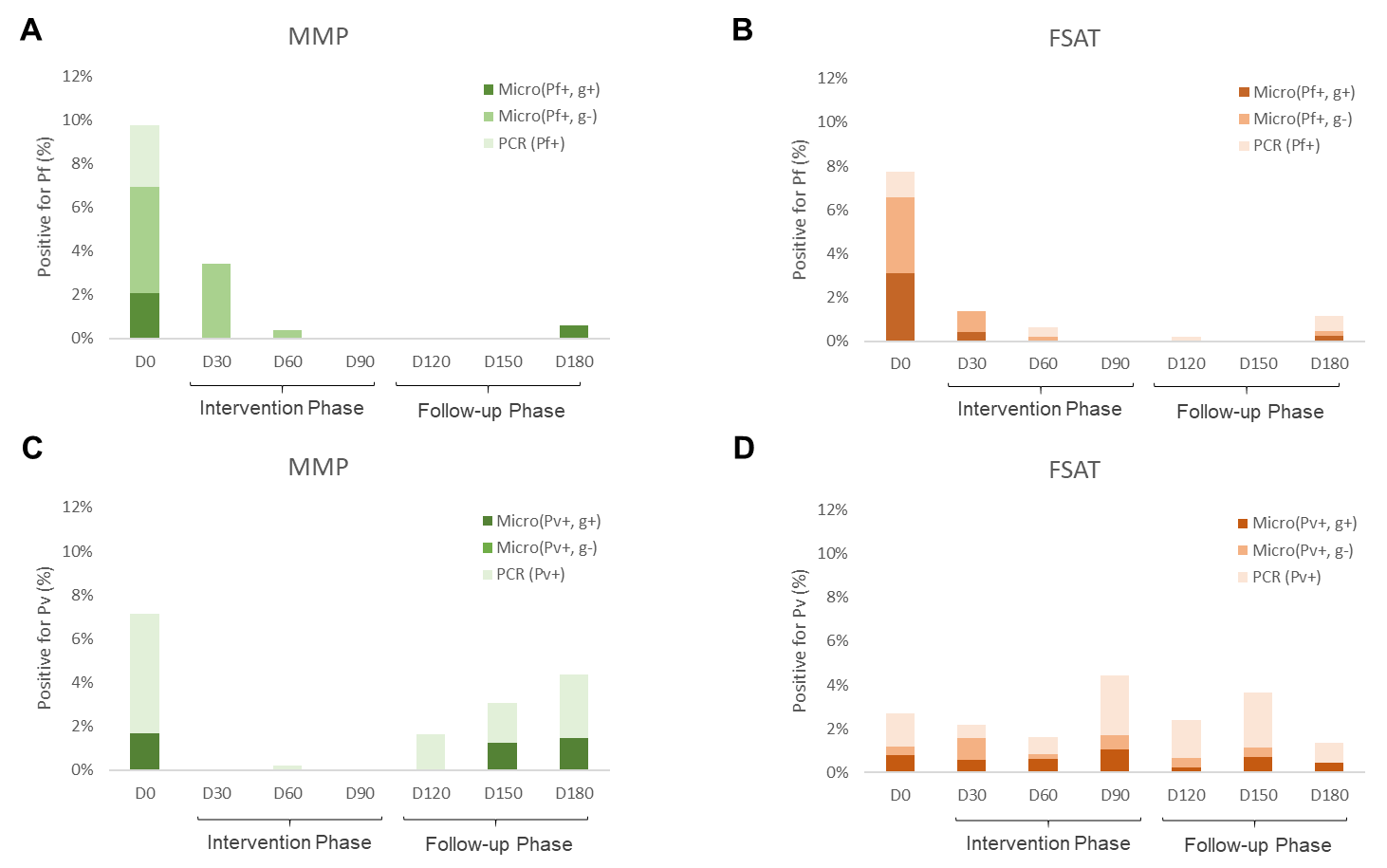


**Supplemental Figure 1. Microscopic gametocytemia by treatment arm for *Pf* and *Pv*.** Presence (g+) or absence (g-) of gametocytes by microscopy was assessed for each individual at each time point. At least 200 oil immersion fields were examined on a thick blood smear before it was considered negative. Panels **A.** and **B.** compare rates of microscopic gametocytemia between the FSAT and MMP cohorts for *Pf* gametocytes, while panels **C.** and **D.** compare rates for *Pv* gametocytes. Overall rates of infection by PCR are indicated by superimposed tan (MMP) or salmon (FSAT) colored bars.

## Recurrent *Pv* infection during study follow-up

Nearly all cases of malaria recurrence (26/30, 86%) after the first monthly follow-up were due to *Pv* malaria (**Fig 3C and D**). There were 13 *Pv* recurrences in FSAT treatment arm and 13 cases in MMP. These *Pv* recurrences may represent relapses or new infections. Logistic regression revealed that recurrence of *Pv* in MMP was associated with diagnosis of *Pv* on enrollment (p<0.001) and lower weekly PQ dose (<5mg/kg/total dose; p<0.001). Among the volunteers with at least one episode of *Pv* malaria, the cumulative risk of *Pv* recurrence (Kaplan-Meier analysis) was 8% in MMP arm and 17% in FSAT within 150 days of follow up (Supplemental **Figure 1A**). There were three notable distinctions between *Pv* recurrences in the MMP and FSAT treatment arms. First, in the MMP arm, 100% (13/13) of cases of *Pv* recurrence were in individuals that tested positive for *Pv* at enrollment which was significantly higher than the 31% (4/13) of cases in FSAT (p < 0.001). Second, in the FSAT treatment arm, subjects wearing sITUs were approximately three times as likely to experience a *Pv* recurrence compared to subjects wearing ITUs (p < 0.01), while in the MMP arm there was no significant difference with respect to uniform. Third, the mean interval between the first and second *Pv* infection was significantly longer for MMP, at 166 days (s.d. 23 days) than for FSAT, at 108 days (s.d. 31 days) (p < 10^-4^

**
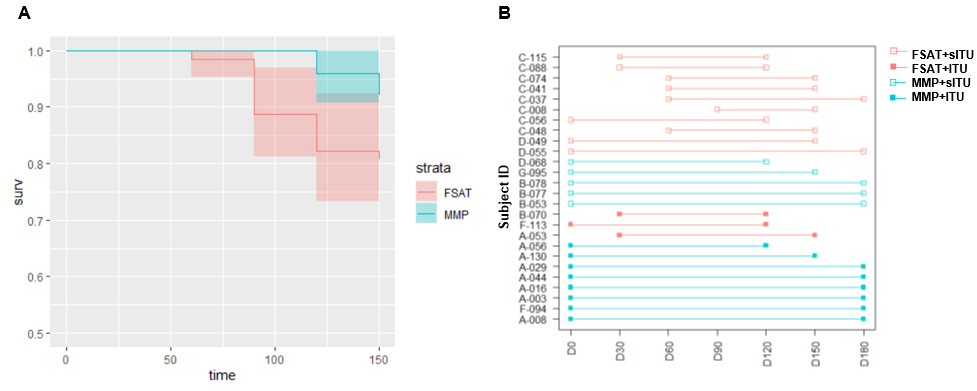
**

**Supplemental Figure 2. Recurrent *Pv* infections in subjects with at least one *Pv* infection.** A) Survival curve to a second *Pv* infection from the first *Pv* infection for all subjects with at least one documented *Pv* infection in the FSAT (red) and MMP (blue) treatment arms. B) Time interval between observing the first and second Pv infection for all subjects that had two documented *Pv* infections in the study. MMP (blue) and FSAT (red) treatment arms are shown as well sITU (open symbol) and ITU (closed symbol).
